# Immediate implant placement influenced by musical flow: a prospective randomized controlled clinical trial

**DOI:** 10.1186/s12903-024-04366-8

**Published:** 2024-05-28

**Authors:** Lorenzo Esteban Pellicer, José Luis Martínez Rubio, Elisabeth Casañas, Antonio Conde Villar

**Affiliations:** 1 Department of Clinical Dentistry, Faculty of Biomedical and Health Sciences, Universidad Europea de Madrid, Plaza Francisco Morano s/n, Madrid, 28005 Spain; 2 School for Doctoral Studies and Research, Universidad Europea de Madrid, Madrid, Spain; 3 Department of Psychology, Faculty of Biomedical and Health Sciences, Universidad Europea de Madrid, Madrid, Spain

**Keywords:** Dental implants, Immediate dental implant loading, Dental anxiety, Music therapy

## Abstract

**Background:**

The purpose of this study was to test how musical flow using baroque (BM) and classical era music (CM) as a non-pharmacological therapy can control anxiety and pain levels among patients undergoing IPI (Immediate post-extraction implants).

**Methods:**

78 patients who required an IPI were enrolled in this randomized clinical trial. Each patient was assigned to one of the three experimental groups with a simple randomization: Group I (*n* = 26) listened to BM; Group II (*n* = 27) listened to CM; and Group III (*n* = 25) did not listen to music and was the control group (C). The physiological dependent variables analyzed were systolic blood pressure (SBP), diastolic blood pressure (DBP), heart rate (HR) and oxygen saturation (SpO2). The psychological dependent variable analyzed was modified dental anxiety scale (MDAS) and visual analogue scale (VAS), measured before and after surgery. In all cases, the level of statistical significance was set at *p* < 0.01.

**Results:**

Statistically significant differences were found in the SBP decrease in the CM group (*p* = 0.001, CI = 1.9716–6.5840) and the BM group (*p* = 0.003, CI = 1.4450–6.4396). Anxiety levels during the intervention decreased in both groups that listened to music: BM group (*p* = 0.002, CI = 0.645–2.662) and CM group (*p* = 0.000, CI = 1.523–3.884).

**Conclusions:**

Patients undergoing IPI placement surgery can register lower levels of SBP when listening to BM and CM than patients who were not exposed to the musical flow, improving their anxiety levels.

## Background

A patient’s appearance is negatively impacted by tooth loss in the anterior maxillary region, putting their hard and soft tissues at risk [1, 2]. Immediate implant placement (IIP) using a flapless approach in the esthetic region is a surgical treatment that yields favorable outcomes [3, 4] and is frequently utilized, preserving both hard and soft tissues while preserving the patient’s esthetics [5, 6]. Compared to delayed implant placement, this is a minimally invasive approach that increases patient comfort and shortens the length of treatment.

However, many patients who need this kind of treatment exhibit rising levels of anxiety and fear, which can reach 76% [7, 8]. It may be challenging to carry out the treatment properly.

Various anxiolytic drugs, such as benzodiazepines and even distraction techniques, have been used as alternative therapies to reduce anxiety, but the results are mixed [8, 9]. . As a safe and noninvasive therapy, music has been shown to aid in the management and control of anxiety during several surgical procedures [9, 10]. However, there is currently no effective musical style for this kind of surgery to lessen patients’ intraoperative fear and anxiety.

Music has proven to be an effective tool in the management and control of anxiety during some surgical procedures [9, 10] and is a safe and noninvasive therapy. However, to date, there is no suitable musical flow in this type of surgery to reduce patients’ intraoperative anxiety and fear [11]. The purpose of this study was to test how musical flow using baroque (BM) and classical music (CM) as alternative therapies can control anxiety and pain levels among patients receiving IIP.

## Methods

### Study design

A prospective randomized controlled clinical trial (RCT) that was double-blind, parallel, and simple to randomize.

### Study population

The sample was composed of all subjects who came to the European University of Madrid´s clinic in Spain between February 2021 and May 2022 for evaluation and management and required the placement of an immediate implant in the aesthetic zone between teeth 14 and 24. The European University of Madrid’s Ethics Committee granted approval (code CIPI/21/005) to the protocol of the study, and this registered in ClinicalTrials.gov the 22/09/2021 with identification NCT05052034.

The inclusion criteria were that the participants were older than 18 years and that Spanish speakers who needed to have a tooth extracted between tooth 14 and 24, as well as an immediate implant placed, were included in the study sample: all the teeth to be extracted had adequate conditions for the procedure, type of extraction socket I or II, as stated by Elian et al. [12], and all had a class I socket with enough palatal bone left for the surgery to be performed properly (type IIc sockets with more significant apical hard tissue involvement were discarded in favor of type IIa and type IIb type II sockets [13]).

Patients who required bone regeneration and flap-lifting surgery and were unable to undergo a flapless procedure were excluded. Additionally, all patients who required a connective tissue graft the same day as surgery were excluded because they had a thin gingival phenotype. All patients with mental illness (dementia), psychiatric disorders, anxiolytic therapy, visual or motor loss, or any other disability that rendered it impossible to complete the provided forms, as well as the visual analog scale (VAS), were also excluded. Patients receiving medical care that might conflict with the anesthesia used for the oral surgery procedure were also excluded.

### Surgical procedure

The upper arch’s canines, first premolars, lateral incisors, and central incisors all received single-tooth implants following extraction [14]. Patients who required any kind of connective tissue graft surgery to improve the peri-implant phenotype were rescheduled for a second surgical phase [15].

The same technique was used for each group. Prior to IIP, the usual diagnostic tests were conducted. Articaine at 4% and epinephrine at 1/200.000 were always used to anesthetize the operative region by mucosal infiltration. More than 4 carpules were never used in total. The tooth to be removed was then luxated after the supracrestal gingival fibers were severed with a 15c scalpel.

**Fig. 1:**
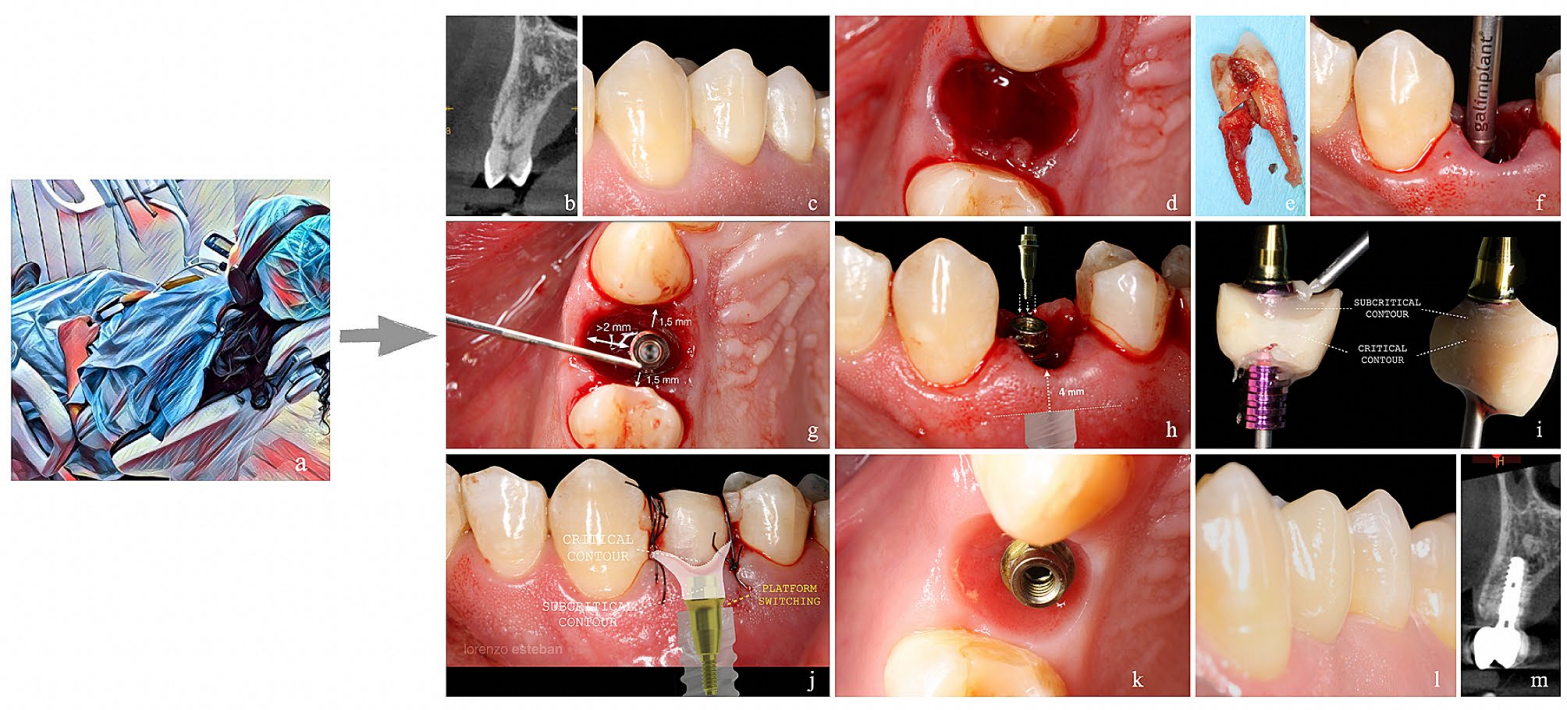
Surgical procedure of IPI performed on all patients in the study. **a**: image of monitored patient under musical intervention **b**: initial CBCT; **c**: intraoral image of tooth 1. 4; **d**: post-extraction socket; **e**: extracted tooth with vertical fracture; **f**: insertion of the first surgical drill; **g**: three-dimensional positioning of implant using a flapless technique; **h**: placement of an esthetic anti-rotational straight abutment; **i**: customization of immediate provisional; **j**: immediate provisional prosthesis; **k**: pink esthetic and emergence profile; **l**: intraoral image of definitive prosthesis; **m**: final CBCT

Dental implants were positioned subcrestally, 3–4 mm from the upcoming gingival edge, according to Linkevicius et al., 2009^16^. A 5/0 monofilament suture was used to close the wound after performing flapless bone regeneration crestal to the vestibular gap. (Braun, Barcelona, Spain). Gonzalez-Martin et al. [16] showed how instantaneous provisionalization was carried out when a primary stability > 25 Ncm (Newton-centimeter) was attained. When adequate primary stability could not be obtained, a Maryland-style temporary bridge was cemented. This had no effect on the study’s findings in any manner (Fig. 1).

### Musical intervention and data collection methods

The patients were randomly divided into three groups: Group I (*n* = 26): Patients who listened to baroque music (BM), Group II (*n* = 27): Patients who listened to classical music (CM) and Group III (*n* = 25): Patients who did not listen to music and composed the control group (C).

After the signing of informed consent form each patient received a brief discussion of potential problems and the opportunity to speak with the physician directly during surgery if needed. Each music group was blinded; any patient could leave the study and cease listening to the music at any time.

The data were randomized, quantified, and gathered by a calibrated, blinded dental examiner. Additionally, every surgery was conducted by the same surgeon, which minimized the impact of various subjective factors that might modify or change the patient’s level of intraoperative anxiety.

Music was heard using Sony WH-CH510 headphones (Sony Corporation, Tokyo, Japan), which were connected to an iPhone X (Apple, California, USA) that was also running the Spotify app. J. Pachelbel, A. Vivaldi, J.S. Bach, and T. Albinoni were heard by the BM group, while W.A. Mozart was heard by the CM group. The average volume was always consistent and never exceeded 60 Db.

The Modified Dental Anxiety Scale (MDAS) [17] was used to measure the participants’ first levels of anxiety. The key predictive factors for surgical operation were the following vital signs: heart rate (HR), oxygen saturation (SpO2), systolic blood pressure (SBP), and diastolic blood pressure (DBP). Due to their variability, each of these metrics was recorded twice sequentially to increase accuracy. An OMRON M2 blood pressure monitor (Omron Healthcare, Kyoto, Japan) was used to measure SBP, DBP, and HR. A Lovia Lox100A pulse oximeter (Noto-Tech Electronics, Shenzen, China) was used to measure oxygen saturation. A Lovia Lox100A pulse oximeter (Noto-Tech Electronics, Shenzen, China) was used to measure oxygen saturation.

The initial vital signs were recorded after the patient had been placed in the dentist chair and before the intervention began. Then, after the anesthesia was administered (second recording of constants), the patient started to listen to music chosen at random. The tooth was luxated and removed after confirming the required anesthetic effect, and the alveolus was thoroughly curettaged. The initial surgical drill was then used to reoperate the bone bed (third recording), and the fourth recording was performed after the procedure was finished. Finally, the patient completed the VAS as previously described [11], and the headphones were removed. The patients then answered the following questions (Table 1): Did you find the music relaxing? would you have selected a different style of music? and would you like to listen to music during your subsequent visits?. The patient was then given standard postsurgical instructions.

**Table 1:** Patient response on listening to music during surgery

		Yes	No	DK/NA	*p* value
Do you think the music helped you relax?	Baroque Music	23	3	0	0.000*
	Classicism Music	23	1	3	0.000*
	TOTAL	46	4	3	0.000*
Would you have chosen another kind of music?	Baroque Music	5	20	0	0.003*
	Classicism Music	9	15	4	0.221
	TOTAL	14	35	4	0.003*
For future visits, would you prefer to listen to music again?	Baroque Music	24	1	1	0.000*
	Classicism Music	25	1	1	0.000*
	TOTAL	49	2	2	0.000*

DK don´t know; NA no answer; * (*p* < 0.01)

### Data analysis

The dependent variables were divided into physiological variables and psychological variables. The physiological dependent variables analyzed were systolic and diastolic blood pressure, heart rate and oxygen saturation, which were recorded at four different times during surgery.

The quantitative variables are described using the median and interquartile range [Q1–Q3] after confirming that they did not follow a normal distribution (Shapiro–Wilk test). The qualitative variables are described using absolute frequencies (n) and relative frequencies (%).

ANOVA was used to compare each of these variables among the three test groups. The psychologically dependent variable analyzed was the degree of anxiety, measured by the self-completed modified dental anxiety scale (MDAS) and VAS (measured before and after surgery). Paired t tests were used to compare the degree of anxiety before and after surgery. For comparisons between qualitative variables, the chi-square test was used. In all the cases, the level of statistical significance was set at *p* < 0.01. The statistical analysis was performed using the IBM SPSS version 27 package program (IBM Software).

### Ethics approval

All participants were informed about the objectives of the study and the protection of the privacy and confidentiality of their data in accordance with regulations (EU) 2016/67 of the European Parliament and of the European Council 27th April 2016 regarding the protection of personal data, its processing and free movement. All the data were collected anonymously. Participants were informed that they could leave the study at any time. All participants gave their informed consent regarding inclusion. No compensation was provided for participation.

The Helsinki Declaration of the World Medical Association (version VI, 2002), as well as additional Spanish legal requirements, was followed in the planning and execution of this study.

## Results

**Fig. 2:**
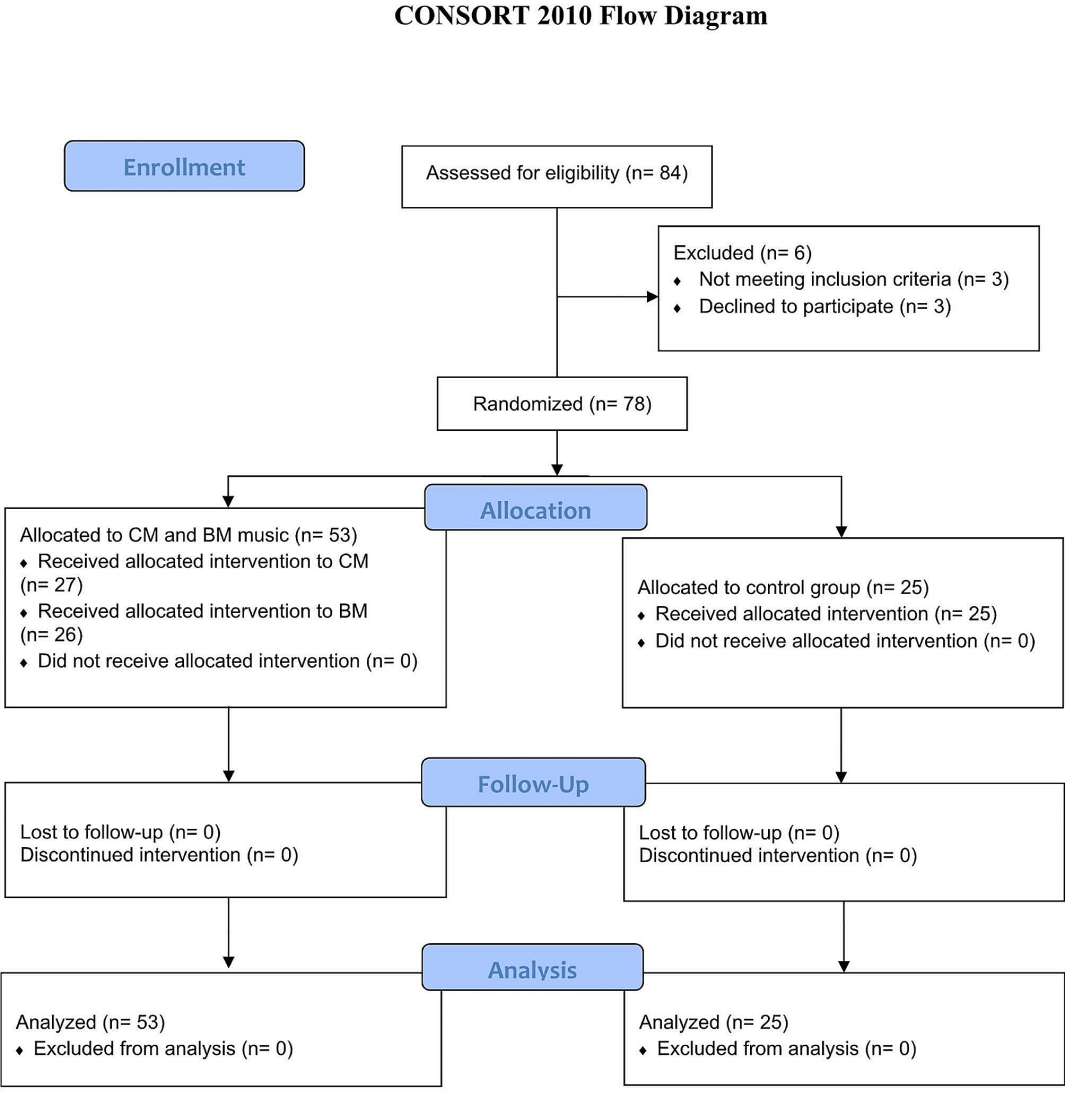
CONSORT flow diagram for trial participation

Three of the 84 patients declined to participate in the study, and three additional patients were excluded because they failed to satisfy any of the requirements. The research population sample consisted of all patients who required immediate implant placement between teeth 14 and 24. The final study population sample included 78 patients, which was conducted between February 2021 and May 2022 (Fig. 2).

**Fig. 3:**
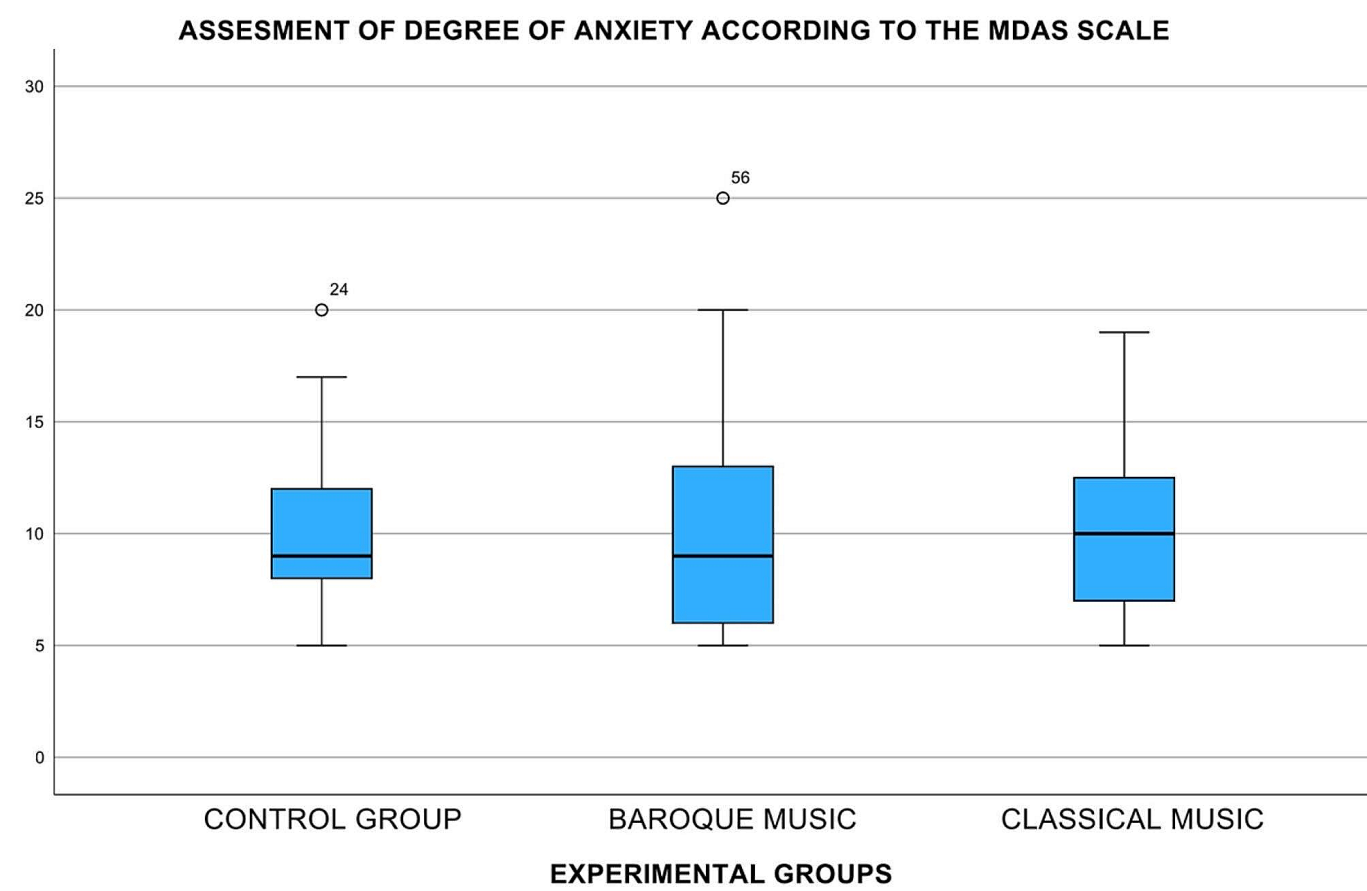
Box plot represents the degree of pre-surgery anxiety by group, showing a homogeneous sample. The *p*-values did not have a statistically significant difference (*p* = 0.831)

**Fig. 4:**
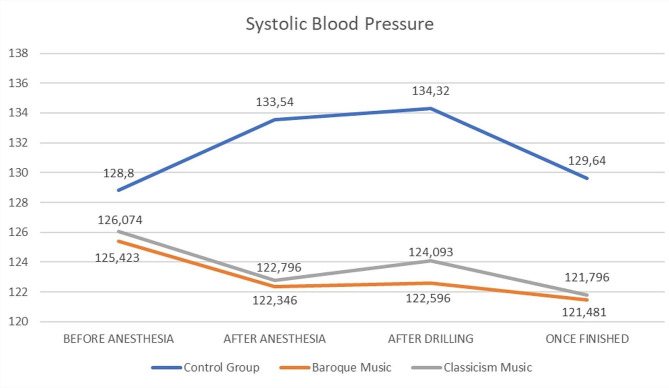
Comparison of mean systolic blood pressure values during immediate dental implant placement surgery between groups. Statistically significant results were found in the decrease of SBP in CM and BM group (*p* = 0.001 and *p* = 0.003, respectively)

The patients had a mean age of 45.7 ± 15.2 years, 47.4% were men, and 52.6% were women. When comparing the MDAS scores before the intervention among the three test groups (groups I, II and III), no significant differences were observed (*p* = 0.831, CI = 9.16–11.17) (Fig. 3). No differences were found for sex (*p* = 0.358) or age (*p* = 0.473) (Table 2). There were also no differences in the SBP before starting treatment between the groups (*p* = 0.634; F = 0.459), which could be considered homogeneous. Significant differences in SBP decrease were found among the three test groups after anesthesia (*p* = 0.005; F = 5.808) and at mid-term treatment (*p* = 0.006; F = 5.478). A decrease in the mean SBP during treatment occurred in group II (*p* = 0.001, CI = 1.9716–6.5840) and group I (*p* = 0.003, CI = 1.4450–6.4396) (Fig. 4). No differences were found in group III (*p* = 0.536, CI=-3.6037-1.9237). There were no statistically significant differences found in DBP, SpO2 or HR.

**Table 2:** Demographic data

		Anxiety Level							
		Control Group (C)		Baroque Music (BM)		Classicism Music (CM)			
		M	SD	M	SD	M	SD	*p* value	
Gender	Male	8,4	3,134	10,5	6,149	9,77	5,15	0.358	
	Female	10,73	4,317	9,67	3,962	11,29	3,221		
	*p value*	0.386		0.691		0.364			
Age	≤ 45	9,85	3,436	8,71	4,427	11,31	3,816	0.473	
	> 45	9,75	4,673	12,78	5,674	9,86	4,639		
	*p value*	0.954		0.054		0.386			
Couple	No	9,07	3,385	8,67	3,708	13,88	4,324	0.998	
	Yes	10,73	5,65	10,88	5,754	9,22	3,524		
	*p value*	0.313		0.308		0.008*			

*M* mean value; *SD* standard deviation. * (*p* < 0.01)

The decrease in anxiety levels during treatment was statistically significant in the test groups that listened to music: group I (*p* = 0.002, CI = 0.645–2.662) and group II (*p* < 0.001, CI = 1.523–3.884). There were no differences in group III (*p* = 0.022, CI = 0.192–2.288) (Table 3). No statistically significant differences were found in pain perception between the groups (*p* = 0.590, F = 0.531).

**Table 3:** VAS for anxiety (before & after) and pain between groups

Groups	Anxiety Level Before Treatment		Anxiety Level After Treatment			Pain Level		
	M	SD	M	SD	*P* value	M	SD	*p* value
Control Group (C)	2,88	2,804	1,64	2,234	0.022*	1,12	2,007	0.590
Baroque Music (BM)	3,31	2,95	1,65	1,958	0.002*	1,15	1,461	
Classicism Music (CM)	4,41	3,445	1,7	1,996	0.000*	0,74	1,347	
*p value*	0.188		0.993					

VAS Visual Analog Scale; M mean value; *SD* standard deviation. * (*p* < 0.01)

Moreover, there was no significant difference in anxiety reduction between the BMs and CMs groups (*p* = 0.172, CI=-0.471-2.570).

Patients in the two groups who underwent auditory listening felt that the music helped them relax (*p* < 0.001), and they preferred to listen to music again at their next visit (*p* < 0.001) (Table 1).

## Discussion

Immediate implant treatment using a flapless technique [2–5] and immediate provisionalization limit bone resorption [18, 19] and improves soft tissue stabilization, improving the patient’s pink aesthetics [20] and gingival phenotype [15].

However, performing double surgery with simultaneous tooth extraction and placement of dental implants may involve a high level of anxiety in patients [10, 21].

The latest studies encourage the implementation of musical flow in dental offices using certain types of music in treatments that generate anxiety, such as dental implants, where satisfactory results are achieved in reducing the level of anxiety in patients in line with the findings of this study [11, 22].

Listening to music can influence both psychological and physiological aspects and even promote neuronal neuroplasticity [23]. The management and control of anxiety and pain levels experienced by patients undergoing IIP is a basic aspect that dentists and dental staff should be aware of.

Several published articles have shown good results using musical hearing in patients undergoing dental extractions [10, 24–27]. Evidence in patients receiving dental implants is scarce [11, 21], and this study provides new knowledge in this regard.

The effect of music on vital signs has been studied, but the mechanism underlying the reduction in blood pressure in patients receiving these treatments is still unclear. In a systematic review by Monteiro et al. [28]. , they found only one study in which there was a statistically significant decrease in systolic blood pressure. The present study adds to the scientific evidence of new favorable results on the statistically significant decrease in systolic blood pressure in patients undergoing tooth extraction and implant placement, as this is the first study of this type of combined treatment.

During oral surgery, the heart rate increases [29]. Classical music seems to reduce heart rate [30], especially at the time of anesthesia administration [31], although the present study found no statistically significant differences.

Likewise, several authors have concluded that listening to music can even positively affect the perception of pain during surgery [32, 33], in contrast to the findings of that study, where no statistically significant differences were observed.

Some limitations of this study could be the number of songs used. As a follow-up study, we recommend a multicenter study, including patients requiring connective tissue grafting and evaluating the influence of a third surgery at the same time. In addition, it would be very interesting to test neuronal neuroplasticity during this type of surgery in patients who are listening to music.

## Conclusion

A lower level of anxiety during the IIP was reported in Spanish patients who listened to classical and baroque music using headphones. The addition of musical flow in an individualized way to each patient in dental clinics is a useful therapy for reducing anxiety and fear in patients and incorporates the possibility of monitoring vital signs, mainly in patients at increased risk of cardiovascular disease.

## Data Availability

The datasets used and analyzed during the current study are available from the corresponding author upon reasonable request.
